# Strength-referenced competence support in physical education for twice-exceptional students with physical disabilities

**DOI:** 10.3389/fpsyg.2026.1980868

**Published:** 2026-10-20

**Authors:** Yasir A. Alsamiri, Omar A. Alsamani, Majed A. Alsharidah, Seham D. Alfaidi

**Affiliations:** 1 Department of Education, Islamic University of Madinah, Madinah, Saudi Arabia; 2 Department of Special Education, College of Education, University of Hai'l, Hail, Saudi Arabia; 3 Department of Curriculum and Instruction, College of Education, Prince Sattam Bin Abdulaziz University, Alkharj, Saudi Arabia; 4 Department of Psychology, Hafr Al Batin University, Hafr Al Batin, Saudi Arabia

**Keywords:** assessment alignment, basic psychological needs, physical disability, physical education, self-determination theory, strength-referenced competence support, twice-exceptionality

## Abstract

Physical education (PE) can make motor performance visible while leaving advanced tactical or analytical capacities unrecognized. For twice-exceptional students with physical disabilities, accessible participation may coexist with insufficient challenge, while recognition of advanced ability may accompany restricted movement. Drawing on Self-Determination Theory and Basic Psychological Need Theory, we develop strength-referenced competence support as an instructional principle. The principle connects evidenced PE-relevant advanced capacity with appropriate challenge, accessible assessment evidence, and substantive recognition, and adapted motor learning. An assessment-and-recognition model specifies how these instructional conditions enter established motivational pathways. Seven propositions examine what changes when access, challenge, recognition, or student influence improves, including a tentative extension to later activity. We distinguish this principle from related educational approaches while identifying conditions under which they provide equivalent support. Its contribution lies in conditional predictions about which learning requirements remain unmet after an adaptation. Comparative tests must establish whether these distinctions improve explanation beyond general teaching measures; familiar need–motivation associations alone are insufficient. The predictions require direct empirical evaluation.

## 1 Introduction

For a twice-exceptional student with a physical disability, an inclusive physical education (PE) lesson may solve an access problem while leaving a learning problem unresolved. A student may participate with adapted equipment yet have no opportunity to exercise advanced tactical understanding; another may contribute sophisticated game analysis but spend the lesson outside movement activities.

The instructional problem concerns both the evidence accepted for learning and the level of challenge offered. An accessible task can reveal understanding while still providing little opportunity to extend it. Research in general PE populations links psychological need experiences to motivation (Vasconcellos et al., 2020; White et al., 2021). Here, we examine how the fit between a learner’s capacities and the task may shape these experiences.

Twice-exceptionality concerns the co-occurrence of high ability or advanced potential with disability (Foley-Nicpon et al., 2011; Reis et al., 2014). Its literature has focused substantially on learning disabilities, attention difficulties, and autism spectrum conditions, with considerable variation in identification and support (Beckmann and Minnaert, 2018; Rizzo et al., 2025). The less understood intersection of advanced ability, physical disability, and PE allows us to examine how support for one dimension of a learner’s profile may leave another unsupported.

Our contribution centers on ability–performance misalignment and strength-referenced competence support. Misalignment occurs when the evidence accepted for an intended PE learning outcome under-represents relevant capacity because the required performance channel is inaccessible or too restrictive. Strength-referenced competence support connects appropriate challenge, accessible evidence, and substantive recognition while supporting motor learning. The assessment-and-recognition model specifies which provision changes should address unmet learning requirements. Self-Determination Theory (SDT), especially Basic Psychological Need Theory (BPNT), explains the proposed need experiences and motivational consequences. Within that explanation, we specify instructional conditions and conditional predictions.

Universal design for learning (UDL) recognizes that learners vary and therefore offers options for engagement, representation, and action and expression, alongside appropriate challenge and feedback (CAST, 2024; Lieberman et al., 2020). Such options can preserve demanding tactical problems while varying how learners show understanding and how movement is supported. Together with mastery-oriented assessment, strengths-based education, and differentiated instruction, UDL informs the proposed provision. Strength-referenced competence support identifies the alignment to examine for each learner.

In BPNT, competence satisfaction refers to the subjective experience of effectiveness, which is distinct from assessed ability or motor performance (Ryan and Deci, 2017). We therefore ask: how can PE support this experience for twice-exceptional students whose demonstrated advanced understanding requires greater conceptual challenge and whose physical disabilities require adapted movement opportunities?

We begin with motivational theory and the twice-exceptionality lens, then examine assessment, recognition, and adaptation. We develop seven propositions to guide practice and research, then consider the scope and limitations of the account.

## 2 Theoretical foundations

### 2.1 Basic psychological needs

Within SDT, BPNT defines autonomy as experiencing volition, competence as experiencing effectiveness, and relatedness as experiencing connection and acceptance (Deci and Ryan, 1985; Ryan and Deci, 2017). These needs are universal, although the conditions that support them may vary across learners and settings (Ryan and Deci, 2020). In this account, advanced ability is relevant because learning opportunities may or may not match a learner’s capacities; it does not represent a stronger or additional psychological need.

Evidence from general PE populations links need satisfaction with autonomous motivation and positive engagement outcomes (Standage et al., 2005; Vasconcellos et al., 2020). Vasconcellos et al. (2020) also identify teacher involvement in autonomy and competence experiences and both teacher and peer involvement in relatedness. These findings support examining classroom and peer conditions together; their predominantly associational basis does not establish the proposed causal processes for the target population.

### 2.2 The satisfaction–frustration asymmetry

BPNT distinguishes low need satisfaction from active need frustration: limited fulfillment does not by itself establish that a need is being thwarted (Bartholomew et al., 2011; Chen et al., 2015; Vansteenkiste et al., 2020). Feeling controlled, experiencing failure or inadequacy, and feeling rejected concern frustration of autonomy, competence, and relatedness, respectively. These experiences are associated with ill-being and maladaptive functioning beyond the absence of positive experiences.

The established bright and dark pathways can coexist: a student may experience choice while encountering an inaccessible assessment, or recognition of advanced analysis alongside exclusion from physical participation. In Section 6, we examine how instructional conditions may shape these mixed experiences.

Evidence from PE supports the distinction. Perceived autonomy-supportive and controlling teaching relate to students’ motivational experiences through partly unique pathways rather than as opposite poles of a single dimension (Haerens et al., 2015). Person-centered study identifies student profiles combining need-supportive and need-thwarting perceptions in ways a single bipolar dimension cannot represent (Burgueño et al., 2024).

### 2.3 Empowering and disempowering motivational climates

Duda's (2013) account contrasts empowering climates, which combine task involvement, autonomy support, and social support, with disempowering climates marked by ego involvement and controlling practices. Studies in PE and teacher education connect these climates with motivational experiences and intentions (Granero-Gallegos et al., 2023, 2024; López-García et al., 2023). In this account, they serve as contextual variables, not evidence of twice-exceptionality itself.

Classroom climate must also be considered in relation to the learner’s task. Two students in the same class may need different conceptual challenges; examine success criteria and access arrangements against each learner’s demonstrated capacity.

The circumplex distinction between autonomy support, structure, control, and chaos (Aelterman et al., 2019; Ahmadi et al., 2023) provides a useful way to describe how provision is organized. When advanced learning is combined with adapted movement, structure refers to clear goals and feedback in both domains, while autonomy refers to the student’s influence over how those domains are combined.

### 2.4 Achievement goals and expectancy-value

Achievement goal theory distinguishes mastery orientations, which emphasize learning and improvement, from performance orientations, which emphasize comparison with others (Ames, 1992; Elliot and McGregor, 2001; Nicholls, 1989). Review evidence links mastery goals and climates in PE to desirable motivational and social outcomes (Jaitner et al., 2019). Our concern is the domain to which mastery criteria are applied: progress in an adapted motor task can be valuable while leaving advanced tactical reasoning unchallenged.

Situated expectancy-value theory proposes that motivation depends on expected success, task value, and perceived cost in a given context (Eccles and Wigfield, 2020). A twice-exceptional learner may value strategic problem solving but anticipate few opportunities to demonstrate it when assessment is dominated by motor execution. Thus, low engagement does not necessarily indicate a lack of relevant capacity. Nor should every available strength be treated as something the student wants to use in PE.

## 3 Twice-exceptionality as a conceptual lens

### 3.1 Definition and its difficulties

Twice-exceptional learners demonstrate high achievement or advanced potential in one or more domains alongside disability (Alsamani, 2026b; Reis et al., 2014). High ability concerns capacity or potential within a domain; sport knowledge concerns understanding of sport and movement; motor competence concerns movement execution; perceived competence concerns the student’s judgment of task-specific capability. These constructs can relate without being interchangeable. Masking and identification difficulties are recognized in the twice-exceptionality literature (Alsamani et al., 2023; Baldwin et al., 2015; Foley-Nicpon et al., 2011), but an identification label alone cannot establish advanced PE-relevant capacity.

Systematic reviews stress the heterogeneity of this population and the need for personalized support, while also noting the limited evidence base and inconsistent comparison groups (Beckmann and Minnaert, 2018; Rizzo et al., 2025). These limitations call for a cautious conceptual analysis rather than broad empirical generalization.

### 3.2 Why the conjunction is informative

Inaccessible assessment, competence frustration, exclusion, and the benefits of student choice can all occur without advanced ability. We use the twice-exceptionality lens to ask whether provision meets both the level of learning a student can undertake and the physical access it requires. An account centered on either dimension may leave the other unspecified. Our aim is to make their joint requirements explicit within PE, rather than propose exclusive psychological mechanisms.

We treat twice-exceptionality as a limiting case to examine whether accessible participation is sufficient evidence of competence support, or recognition of talent sufficient evidence of inclusion. PE is informative because movement is both a learning outcome and a channel through which broader understanding is judged. An alternative response can reveal advanced reasoning without securing movement opportunities; adapted movement can secure access without extending reasoning. Learners facing language or sensory barriers may exhibit comparable limitations. The rationale is curricular tension, not the profile’s rarity or an assumption of greater distress.

Giftedness in another domain does not demonstrate advanced tactical, analytical, or creative capacity in PE. The model makes its strongest predictions when relevant capacity is already demonstrated or becomes visible under accessible instruction. Without such evidence, it supports exploring potential alongside accessible teaching. A student without a gifted designation who shows the same capacity and requires the same access should be expected to follow the same proposed mechanism.

Developmental conceptions of giftedness emphasize opportunities through which potential becomes expressed (Alsamani, 2019; Gagné, 2004; Renzulli, 1978; Subotnik et al., 2011). A conceptual task that appropriately challenges one learner may offer another little opportunity to extend understanding. Therefore, reducing motor demands and increasing conceptual demands may therefore be required together. Advanced ability changes the challenge and evidence needed to recognize learning. It can also make a non-motor role useful to the class, creating a risk that this usefulness becomes a reason to restrict movement (Section 4.3).

Consider two learners with similar physical access requirements and prior learning opportunities: one demonstrates advanced tactical reasoning, while the current tactical task appropriately challenges the other. Adapted equipment may improve access for both while leaving only the first learner underchallenged. With access held constant, increased conceptual demand should address that mismatch. A learner with comparable advanced reasoning but no physical access barrier may need the same challenge without movement adaptations. These comparisons locate the role of advanced capacity in responses to provision, without assuming a uniform group disadvantage or greater-than-additive effects.

Systemic study similarly locates difficulties in the fit between learners’ capacities and educational resources, arguing that separately introduced supports may leave regulatory demands unresolved (Ziegler et al., 2026). Building on this concern with fit, we specify which PE learning evidence counts, how it becomes consequential, and how recognition can coexist with restricted movement. We link these conditions to need experiences.

## 4 Recognizing advanced understanding alongside motor learning

### 4.1 Matching assessment evidence to the learner and the goal

A learner who can solve a tactical problem may still feel ineffective if the lesson offers no usable way to contribute the solution. Competence satisfaction concerns this experience of effectiveness (Ryan and Deci, 2017), distinct from demonstrated ability or judgments of task-specific capability. Assessment must therefore consider how advanced understanding can be expressed and used.

Assessment for this learner must distinguish advanced understanding from motor execution while recognizing learning in both. Speed or accuracy may be valid evidence for a movement goal; an accessible explanation may reveal reasoning relevant to a tactical goal. Either norm-referenced or criterion-referenced assessment can be aligned in this way. The specific concern is whether the response mode and task level together represent the learner’s relevant capacity.

To illustrate the assessment problem, consider a hypothetical student with cerebral palsy affecting gait and upper-limb coordination who also demonstrates advanced tactical understanding of invasion games. If a task claims to assess tactical decision-making but accepts only rapid execution, the student’s reasoning may remain hidden. An accessible explanation or assisted enactment could provide evidence of that reasoning, while a separate adapted criterion assesses motor learning. The proposed motivational mechanism is the student’s experience that relevant effort and understanding cannot count as successful learning. Whether this produces low satisfaction or active frustration depends on the learner’s experience.

P1. Among twice-exceptional students with demonstrated PE-relevant advanced capacities, restricting evidence for a broader learning goal to disability-constrained motor performance is expected to predict greater competence frustration than an accessible, construct-aligned assessment of that goal, beyond differences in motor competence.

P1 identifies a problem shared with other students whose understanding is obscured by an inaccessible response mode. Advanced capacity matters when even an accessible assessment limits the reasoning the learner can demonstrate (P2). For motor learning goals, however, analysis cannot replace evidence of movement. Section 11 sets out tests of P1.

### 4.2 Defining strength-referenced competence support

We define strength-referenced competence support as an instructional principle that coordinates four conditions: evidence of a PE-relevant advanced capacity informs the learning goal; the task provides appropriate challenge for that capacity; an accessible response mode yields valid evidence of learning; and that evidence informs feedback, assessment, or decisions within shared activity. Its PE application also preserves meaningful adapted motor learning. The unit of analysis is provision for a particular learner and learning goal across an instructional sequence. It describes an observable arrangement intended to support competence, rather than a learner trait or the experience of competence satisfaction itself.

“Strength-referenced” identifies evidence for selecting challenge, not a rule for grading presumed ability. Strength means demonstrated or emerging advanced capacity relevant to the PE goal, rather than a preferred activity or relative personal advantage. Evidence of emerging capacity should remain provisional, based on accessible learning opportunities and subject to review over time. Performance should still be judged against explicit learning criteria, while effort, strategies, and progress are recognized.

Substantive recognition means using learning evidence in feedback, assessment, or shared activity. Feedback qualifies when it addresses reasoning against the goal and guides further learning; it may be critical, and a tactical proposal need not be accepted. Generic praise or appointment as team analyst does not by itself qualify. Recognition may be private; public attribution and peer uptake are separate conditions in Section 5. Temporary alternatives to movement should be reviewed in relation to fatigue, access, goals, and preferences.

We position this principle within established approaches, specifying the alignment to examine for a particular learner. Mastery-oriented assessment focuses on learning and improvement (Ames, 1992; Jaitner et al., 2019); strengths-based education develops capabilities (Baldwin et al., 2015; Reis et al., 2014; Alsamani, 2026a); and differentiated instruction responds to learner needs (Tomlinson, 2014). UDL adds challenge, accessible ways to express understanding, and informative feedback (CAST, 2024; Rose and Meyer, 2002). Table 1 sets out these relationships.

**Table 1:** Relationship to established educational approaches.

Approach	Organizing focus	Relation to the proposed principle
Mastery-oriented assessment	Success through learning, understanding, and improvement.	The principle specifies which advanced learning is challenged and recognized alongside movement. It is compatible with mastery criteria and does not grade presumed ability.
Strengths-based education	Recognition and development of capabilities as resources for learning.	Evidence of advanced capacity relevant to physical education informs challenge. Accessible learning evidence must then be used in feedback, assessment, or shared activity; valuing a strength alone is insufficient.
Differentiated instruction	Responsive variation in curriculum, teaching, and assessment for learner needs.	Differentiation can produce the whole arrangement. The principle specifies what to examine in the resulting provision when advanced learning and physical access require coordinated adjustments.
Universal Design for Learning (UDL)	Design for variability through options for engagement, representation, and action and expression.	UDL includes challenge and informative feedback. The principle examines whether the options used by this learner realize the specified alignment while preserving adapted motor learning.

Each approach can fully implement the proposed principle; the distinctions concern its unit of analysis and purpose. Sources: Ames (1992), Jaitner et al. (2019), Baldwin et al. (2015), Reis et al. (2014), Tomlinson (2014), and CAST (2024).

Full implementation requires all four conditions alongside continuing motor learning. This descriptive boundary does not imply that every component is necessary for every benefit or that their combination guarantees need satisfaction. The principle helps identify incomplete provision and the change required. Established approaches meeting the same conditions are expected to be functionally equivalent, with no additional effect from the strength-referenced label.

For example, a learner with evidenced advanced game analysis compares defensive systems, justifies a demanding decision through a diagram, and revises the argument using criterion-based feedback, alongside adapted movement goals. Private feedback can meet the definition even if the team chooses another strategy. A routine diagram task leaves challenge unresolved; demanding analysis filed without use leaves recognition unresolved; a permanent analyst role without movement leaves the PE application incomplete.

P2. Among twice-exceptional students with demonstrated PE-relevant advanced capacities, evidence of advanced learning informing feedback, assessment, or shared activity is expected to improve competence satisfaction beyond providing an equally accessible and challenging task whose evidence remains inconsequential. The added benefit is expected to depend on prior under-recognition; when existing provision already meets these conditions, the strength-referenced label is not expected to add benefit.

### 4.3 The masking risk

A twice-exceptional student may be retained as the team’s analyst because their contribution is useful, while movement adaptations receive less attention. This allocation can mask an unmet learning requirement: recognizing one capacity can obscure limited opportunities in another domain. The risk depends on how the role is organized and is not exclusive to gifted students.

For a student with a physical disability and advanced tactical understanding, examine whether an analytical role complements movement or routinely replaces it, taking account of learning goals, access requirements, and the student’s preferences. Meaningful physical participation remains an entitlement for every student, regardless of gifted identification or tactical contribution.

## 5 Peer recognition and participation through advanced contributions

A twice-exceptional learner’s advanced tactical contribution may be less visible than their motor performance. Teammates might use a solution without knowing who developed it, while readily attributing a missed pass. In P3, we ask whether visible use and acknowledgment of that contribution changes the learner’s experience of effectiveness and belonging.

Research on inclusion in PE shows that participation and peer relationships require attention (White et al., 2021; Block and Obrusnikova, 2007; Coates and Vickerman, 2010; Fitzgerald, 2005; Haegele and Sutherland, 2015; Spencer-Cavaliere and Watkinson, 2010; Wilhelmsen and Sørensen, 2017). It does not demonstrate an effect of advanced ability. Here, independently evidenced advanced understanding is treated as one possible route into shared activity, whose use can be examined. It does not imply a stronger need for belonging or make acceptance conditional on exceptional performance.

P3. For twice-exceptional students whose advanced PE-relevant contributions remain unrecognized, publicly visible motor difficulty is expected to be more strongly associated with concurrent competence and relatedness frustration than when those contributions are visibly used and acknowledged within shared activity.

Recognition and access to play can still diverge. A team may value a learner’s advanced analysis, but selection based on unadapted motor output can exclude them from movement. P4 concerns whether participation rules permit both contributions, beyond peers’ stated appreciation.

P4. Among twice-exceptional students with relevant advanced capacities, selection and success rules that admit only disability-constrained motor contributions are expected to predict reduced meaningful participation even when peers acknowledge those capacities. Rules that admit both advanced contributions and accessible movement are expected to reduce this structural exclusion.

Different settings may recognize different aspects of a twice-exceptional learner’s profile (Baldwin et al., 2015; Foley-Nicpon et al., 2011). A specialist role may bring recognition alongside limited reciprocal interaction or unwanted public attention. Section 11 sets out tests of P3 and P4.

## 6 An assessment and recognition model within established motivational pathways

We now bring the preceding argument together in an assessment-and-recognition model that links misalignment with the proposed instructional response. We ask which learning requirement remains unmet after an adaptation and which experience should improve if that requirement is addressed. The resulting comparisons distinguish arrangements that may otherwise appear equally inclusive.

### 6.1 Specifying instructional antecedents within existing theory

SDT and BPNT explain relations between need experiences and motivation, including mixed experiences of satisfaction and frustration (Haerens et al., 2015; Vansteenkiste et al., 2020). The model specifies instructional conditions within these theories: whether tasks extend relevant capacity, whether accepted evidence represents learning, and whether recognition preserves movement opportunities.

The proposed sequence begins with independently evidenced capacity and the intended learning goal. Task demands determine whether capacity can be extended, response modes whether learning can be demonstrated, and assessment and feedback whether the evidence has consequences. Participation rules separately govern access to shared movement. These conditions are expected to shape effectiveness, volition, and connection within established motivational pathways. They must be observed independently of need reports to avoid defining support by its outcome.

Admitting evidence and using it require different changes. An accessible, demanding analysis task may reveal advanced understanding, yet its product may remain irrelevant to feedback, assessment, or play. Correcting the response mode addresses misalignment (P1); making the evidence count addresses under-recognition (P2). Movement opportunities may require further changes to participation rules (P4).

### 6.2 Conditional predictions from incomplete provision

Responses depend on the learner’s initial relation to the task. Once physical access is secured, increasing conceptual demand should improve experienced effectiveness when the previous task underchallenged evidenced capacity. The same increase may be unnecessary or excessive for a learner who is appropriately challenged. P2 similarly predicts a benefit from recognition when advanced learning was previously disregarded.

Recognition differs across propositions: P2 includes private feedback or assessment; P3 concerns visible peer uptake and acknowledgment; P4 concerns movement access despite acknowledgment. These conditions can change separately.

These configurations enter the familiar motivational pathways. Lack of appropriate challenge may leave competence insufficiently satisfied; devaluation, coercion, or exclusion may actively frustrate needs. Need satisfaction is associated with autonomous motivation and engagement, whereas need frustration may be associated with controlled motivation, amotivation, or withdrawal (Bartholomew et al., 2011; Haerens et al., 2015; Vasconcellos et al., 2020). Controlled motivation and amotivation remain distinct possibilities, not stages in a required sequence. The model therefore does not infer frustration from an incomplete adaptation or reduced movement alone.

### 6.3 Distinguishing the central predictions from inherited relations

P5 predicts different patterns of experienced effectiveness following changes in motor access or provision for advanced learning. Table 2 compares these arrangements, with choice and peer relationships treated separately. The comparison concerns two contexts for the same competence need. Effects may extend across contexts; the prediction concerns their relative pattern.

**Table 2:** Predicted experiences across movement and advanced learning provision.

Motor learning access	Advanced learning provision	Predicted experience	Links
Provided	Appropriate challenge, accessible evidence, and substantive recognition	Opportunities for effectiveness in both movement and advanced learning. Agency and belonging depend on additional conditions.	P2, P5
Provided	Relevant capacity remains underchallenged or unrecognized	Improved effectiveness in movement may coexist with little opportunity to use or extend advanced understanding.	P2, P5
Restricted	Appropriate challenge and recognition are provided	Effectiveness in advanced learning may coexist with restricted movement. Autonomy frustration depends on how the restriction is experienced and negotiated.	P4–P6
Restricted	Relevant capacity remains underchallenged or unrecognized	Limited opportunities in both domains; satisfaction is low or frustration is active, depending on the student’s experience.	P2, P4, P5

These are instructional comparisons, not validated student types. Advanced learning provision combines challenge, accessible expression, and substantive recognition; measure these components separately. Choice and peer participation may vary in every configuration. Assess low satisfaction and active frustration separately, including the learner’s experience of temporary alternatives to movement.

P5. For twice-exceptional students with demonstrated PE-relevant advanced capacities whose initial provision constrains both movement and advanced learning, improvements confined to motor access are expected to improve movement-related effectiveness more than effectiveness in expressing advanced understanding. Improvements confined to advanced challenge and recognition are expected to show the reverse pattern. Provision addressing both is expected to support effectiveness across both domains, without assuming a greater-than-additive effect. These experiences may coexist with other need frustrations when choice or reciprocal participation is restricted.

P6. When motor access and advanced challenge are comparable, participation in choosing and reviewing adaptations is expected to support greater autonomy satisfaction than imposed allocation to the same activities. For twice-exceptional students, this includes a meaningful choice about how advanced contributions and movement participation are combined.

P7. For twice-exceptional students with physical disabilities and PE-relevant advanced capacities, repeated access to both appropriately challenging strength expression and meaningful motor learning is expected to predict sustained physical activity through more favorable need experiences and autonomous motivation, beyond baseline activity, motor competence, and continuing access conditions.

P1–P5 specify the central instructional contrasts. P6 applies an established autonomy mechanism, while P7 tentatively extends the account to later activity. Confirming P6 or familiar need–motivation associations alone would not establish the model’s added value. In Section 11, we outline tests of whether the central contrasts improve explanation and instructional decisions beyond less differentiated accounts.

## 7 Coordinating advanced challenge and physical access

### 7.1 Negotiating challenge and participation

Autonomy-supportive practices can help learners negotiate physical access and conceptual challenge (Ahmadi et al., 2023; Reeve and Cheon, 2021). Advanced capacity changes the decisions involved, including whether demanding analysis supplements or displaces desired movement. P6 applies the general autonomy mechanism to these decisions.

The learner contributes knowledge of preferences, effort, and access barriers, and may offer advanced analysis of a task when that capacity is demonstrated. Neither giftedness nor disability establishes design expertise or an obligation to provide it. Teachers and relevant specialists retain responsibility for instruction, feasibility, and safety.

Agency may involve revising an analytical role, retaining adapted movement, or asking an adult to lead planning. P6 concerns influence over the arrangement at comparable levels of access and challenge, without requiring the learner to design the adaptation independently.

### 7.2 Maintaining learning goals during adaptation

When a learner’s advanced understanding and movement access require different adjustments, adaptation should retain explicit learning goals for each. For example, an analysis task offered during fatigue can extend demonstrated tactical reasoning if it includes a suitably demanding problem and feedback. Simply assigning more written work does not establish advanced challenge, and the temporary task should be reviewed alongside ongoing opportunities for motor learning.

Review which learning opportunity the adaptation preserved or postponed and whether the learner influenced the decision. The learner’s account distinguishes a chosen temporary change from an imposed continuing role; reduced movement alone does not establish frustration.

### 7.3 Using universal design and cooperative tasks

UDL-informed options can keep conceptual complexity distinct from the physical demands of expressing a solution (CAST, 2024; Rose and Meyer, 2002; Lieberman et al., 2020). A diagram, spoken explanation, or assisted enactment can show the intended level of reasoning alongside an adapted movement goal.

Cooperative tasks can give advanced reasoning a meaningful role: peers can examine and use a solution in shared play while the learner participates through an accessible movement role. Reviews provide a rationale for examining such arrangements (Casey and Goodyear, 2015; Fernández-Espínola et al., 2020), but their benefits for this profile require direct evaluation. Evidence should focus on actual use and participation, not simply on assignment to a named role.

## 8 Physical literacy and life skills as psychosocial outcomes

Physical literacy includes motivation, confidence, physical competence, knowledge, and understanding in relation to lifelong physical activity (Cairney et al., 2019; Edwards et al., 2017; Whitehead, 2010). For twice-exceptional students, strong knowledge or reasoning may coexist with constrained motor performance and varied motivation. No single component establishes the others. The model predicts that providing support for both relevant strengths and adapted motor learning may help connect these dimensions, but empirical testing is needed.

Authentic participation may also offer opportunities to practice communication, problem solving, and leadership. The life-skills literature treats transfer beyond the original activity as a process requiring explanation and support (Gould and Carson, 2008; Holt et al., 2017; Pierce et al., 2017). A twice-exceptional student who negotiates an adaptation or explains a tactical solution may exercise such skills, but neither advanced ability nor assignment to a leadership role demonstrates that transfer has occurred.

## 9 Measurement considerations

Measures developed for need satisfaction, need frustration, and motivational regulation provide a starting point, although their settings and validation samples differ (Chen et al., 2015; Haerens et al., 2015; Vlachopoulos and Michailidou, 2006). Their suitability for twice-exceptional students with physical disabilities cannot be assumed. We identify three distinctions that matter for testing the propositions.

First, assess relevant advanced capacity independently of both gifted identification and motivational outcomes, using accessible tasks, developmentally appropriate standards, and information on prior learning opportunities. Knowledge gained through extensive sport exposure should not automatically be treated as evidence of advanced general ability. Assessments should identify misalignment by comparing the stated learning goal, the evidence accepted, and the capacity demonstrated through a valid alternative mode. It is not a raw difference score between intellectual and motor measures. Perceived capability and competence satisfaction should be measured separately, and measurement equivalence should be examined before group comparisons are made.

Second, observe provision independently of need reports. Record the conditions in Section 4.2 for a defined instructional sequence, including the recognition channel: feedback, assessment, or shared activity. These conditions describe provision rather than form an established psychological scale; a total score may conceal a missing component. Student reports should assess effectiveness, volition, and connection separately. For P5, reports of movement and advanced learning should supplement validated measures of competence satisfaction because they address the same need in different contexts.

Third, sampling should distinguish formal identification, demonstrated domain-specific capacity, and emerging potential. Recruitment based solely on gifted labels may miss relevant profiles, while teacher nomination and accessible performance tasks also carry biases. Researchers should report prior opportunities to learn, the criteria used to identify advanced capacity, and the nature of physical access requirements. The construct should not be defined retrospectively by which students benefit from the intervention.

## 10 Implications for practice and teacher education

From the seven propositions, we draw the following implications for instructional decisions. Their effectiveness for twice-exceptional students with physical disabilities requires evaluation.

Assessment alignment (P1). Identify the intended learning goal and permit an accessible response, such as explaining a tactical decision through a diagram, so motor difficulty does not obscure advanced understanding. Retain a separate adapted motor goal where movement is assessed.

Use of learning evidence (P2). Once challenge and accessible expression are appropriate, use the learner’s work in feedback, assessment, or shared activity. For example, criterion-based feedback on a tactical argument can guide revision and make the evidence consequential. Private feedback can qualify under Section 4.2.

Peer recognition (P3). Where motor difficulty is visible but advanced contributions remain unrecognized, enable peers to examine and use the learner’s reasoning. Agree attribution with the student and ask separately about feeling ineffective and excluded. This addresses P3’s proposed recognition mechanism beyond public praise.

Participation rules (P4). If peers value the learner’s analysis but the learner remains outside play, review grouping, scoring, and participation rules. Arrange an accessible movement role and check actual participation; further acknowledgment alone may leave this structural barrier unchanged.

Review of partial support (P5). Check access, challenge, and recognition, then ask separately about effectiveness in movement and advanced learning. Improvement confined to one domain directs attention to provision in the other, following P5’s predicted pattern.

Influence over adaptations (P6). Involve the learner in deciding how analysis and movement are combined, including the duration and review of specialist roles. Check whether preferences shaped the arrangement even when access and challenge are comparable. Choosing adult guidance can also express agency.

Continuity across transitions (P7). Plan with the learner to carry movement adaptations and advanced learning opportunities into later classes or activities. Review need experiences, reasons for continued participation, and actual opportunities. P7 predicts a motivational link; participation alone cannot establish that pathway.

PE, gifted-education, and disability-support staff can coordinate these decisions in one learning plan, drawing on evidence of capacity, access requirements, and student preferences.

Teacher education can use a twice-exceptional learner’s profile to rehearse these decisions. Existing study on need-supportive teaching (Ahmadi et al., 2023; Reeve and Cheon, 2021) and twice-exceptionality (Alsamani et al., 2026; Alsamiri and Alsamani, 2026) can inform this preparation. Teachers can identify the relevant proposition, justify a corresponding change, and review it in light of the student’s experience.

## 11 Implications for research

We propose a research agenda that tests the distinct roles of P1–P7 identified in Section 6.3. Across these tests, preregister comparisons and examine alternative explanations, including prior sport experience, teacher attention, task interest, fatigue, and actual access.

For P1, compare assessments of the same goal that differ in whether valid accessible evidence of advanced reasoning is admitted, keeping content and instructional exposure comparable. Establish capacity beforehand using independent accessible tasks. Distinguish the constructs in Section 9 and record task performance separately from motor competence; a low score does not itself establish frustration. A within-student contrast can test whether frustration changes without motor improvement. Norm-referenced versus criterion-referenced formats alone would not isolate misalignment.

For P2, compare equally accessible and challenging tasks that differ in the use of advanced learning evidence, matching teacher contact, workload, and movement opportunities where possible. Specify the recognition channel and measure remaining differences: a feedback comparison alone cannot establish peer uptake or distinguish the principle from informative feedback. Active comparators using mastery-oriented, strengths-based, differentiated, or UDL-informed provision should match the defining conditions; similar outcomes would support functional equivalence. Improvement over motor-only provision could show practical benefit without distinguishing the principle from existing approaches. Test capacity and prior under-recognition as moderators.

For P3, combine repeated student reports with observations of motor-performance visibility, peer uptake, and attribution of advanced contributions. Distinguish visible difficulty from poor performance, and substantive recognition from friendliness or praise. Test whether recognition moderates associations of visible motor difficulty with competence and relatedness frustration, accounting for baseline peer relationships and preferences about publicity. Student accounts can clarify whether an acknowledged role enables reciprocal interaction. Include appropriately challenging contributions across capacity levels: similar benefits irrespective of capacity and prior provision would support a general recognition mechanism. Cross-subject comparisons should measure visibility rather than assume PE is uniquely public.

For P4, compare task structures that provide different routes to participation while measuring peer attitudes and behavior separately. Observe whether a student whose tactical capacity is already acknowledged gains access to meaningful play when grouping, scoring, or participation rules change. Natural variation or interventions improving access can examine structural effects without deliberately exposing students to exclusion. A structural explanation requires evidence about the rules and how they operate, beyond a general measure of negative climate.

For P5, first establish that movement and advanced learning are both constrained. Motor access, challenge relative to capacity, recognition, student influence, and peer participation should be measured separately. Repeated observations or sequential improvements can compare changes in movement-related effectiveness with changes in advanced-learning effectiveness, while keeping teacher contact and choice comparable where feasible. The predicted difference between changes across domains should be tested directly, rather than inferred from significance in one domain alone. Multilevel analyses can examine within-student changes; person-centered analyses may describe mixed profiles where sample size permits (Burgueño et al., 2024). For P6, adaptation processes should be compared when final access and challenge are similar, while perceived influence is recorded. Voluntary reliance on adult guidance is not equivalent to autonomy frustration.

For P7, follow students across school transitions into later activity. Test whether repeated joint provision predicts subsequent activity through need experiences and autonomous motivation, accounting for baseline activity, motor competence, health, fatigue, and continuing access. Analyze temporal ordering and alternative explanations; an observational indirect association would not establish causation. This extension is less certain than the immediate classroom predictions.

To examine advanced ability, recruit learners with similar physical access requirements across independently assessed PE capacity levels, including twice-exceptional students whose strengths lie outside PE. After comparable access is provided, test whether residual underchallenge and responses to increased conceptual demand vary with capacity and initial task demands. Where feasible, include learners with comparable advanced capacity but no physical access barrier to distinguish challenge requirements from the need to coordinate movement adaptations. Measure prior sport experience, learning opportunities, and initial recognition. These comparisons test conditional responses to provision rather than gifted-group differences alone.

Small samples may first support repeated within-person or replicated single-case comparisons. Capacity-by-provision interactions and profile models require adequate precision before strong conclusions. A null result with imprecise estimates would not establish equivalence. Report disability-related variation and cultural context, and include student voice through accessible formats.

Initial qualitative and observational studies should clarify students’ experiences of underchallenge, recognition, and restricted roles. A subsequent model comparison can retain the same need–motivation relations in both accounts. One predicts need experiences from established measures of autonomy support, structure, and control; the other adds observed assessment alignment, recognition, movement opportunities, and prespecified relations to capacity. Evaluate improvement in predicting need experiences and responses to changed provision, reporting uncertainty. This tests an instructional specification within SDT. Need experiences remain potential mediators of later motivation; a direct effect after adjusting for them is not required.

## 12 Scope and limitations of the analysis

Our analysis draws on motivational theory and adjacent research; it is neither a systematic synthesis nor a validated intervention. The propositions remain untested in the target population, and a detailed application of SDT may require similar distinctions. The value of our account therefore depends on useful conditional predictions and instructional decisions. Precise evidence of no predicted domain-specific contrast, or benefits explained entirely by extra attention or easier work, would weaken its claims.

Applicability depends on demonstrated or emerging PE-relevant capacity and physical access requirements, including among learners outside formal twice-exceptional identification. Disabilities vary in visibility, fatigue, pain, progression, and assistive technology access; advanced potential varies by domain and prior learning opportunity. Cultural and institutional differences in assessment, identification, participation, and preferred recognition may also affect the model’s applicability.

Critical disability studies, embodiment approaches, and intersectional analyses could extend our SDT- and BPNT-based account of institutional power and the social valuation of bodies and abilities. Recognition of advanced contribution must never become a condition for access, dignity, or belonging.

## 13 Conclusion

We have argued that twice-exceptionality makes a specific instructional tension in PE visible: accessible participation may leave advanced learning underchallenged, while recognition of advanced contribution may coexist with restricted movement. Strength-referenced competence support brings together appropriate challenge, accessible evidence, and substantive recognition, and adapted motor learning. It helps identify the unmet requirement for a particular learner and can be implemented through established educational approaches.

The assessment-and-recognition model specifies conditional predictions about which experiences should improve when provision changes. SDT and BPNT explain the motivational processes. Testing these predictions against less differentiated accounts and functionally equivalent practices will determine whether the proposed specification adds explanatory and practical value.
